# Deep learning for determining the difficulty of endodontic treatment: a pilot study

**DOI:** 10.1186/s12903-024-04235-4

**Published:** 2024-05-17

**Authors:** Hamed Karkehabadi, Elham Khoshbin, Nikoo Ghasemi, Amal Mahavi, Hossein Mohammad-Rahimi, Soroush Sadr

**Affiliations:** 1 Present Address: Department of Endodontics, Dental School, Hamadan University of Medical Sciences, Hamadan, Iran; 2 Faculty of Dentistry, Zanjan University of Medical Sciences, Zanjan, Iran; 3 Topic Group Dental Diagnostics and Digital Dentistry, ITU/WHO Focus Group AI on Health, Berlin, Federal Republic of Germany; 4 Dental School, Hamadan University of Medical Sciences, Shahid Fahmideh Street, PO Box 6517838677, Hamadan, Iran; 5 Department of Endodontics, Dental Research Center, Hamadan University of Medical Sciences, Hamadan, Iran

**Keywords:** Deep learning, Case difficulty, Endodontics, Classification, Regression, Self-supervised learning

## Abstract

**Background:**

To develop and validate a deep learning model for automated assessment of endodontic case difficulty from periapical radiographs.

**Methods:**

A dataset of 1,386 periapical radiographs was compiled from two clinical sites. Two dentists and two endodontists annotated the radiographs for difficulty using the “simple assessment” criteria from the American Association of Endodontists’ case difficulty assessment form in the Endocase application. A classification task labeled cases as “easy” or “hard”, while regression predicted overall difficulty scores. Convolutional neural networks (i.e. VGG16, ResNet18, ResNet50, ResNext50, and Inception v2) were used, with a baseline model trained via transfer learning from ImageNet weights. Other models was pre-trained using self-supervised contrastive learning (i.e. BYOL, SimCLR, MoCo, and DINO) on 20,295 unlabeled dental radiographs to learn representation without manual labels. Both models were evaluated using 10-fold cross-validation, with performance compared to seven human examiners (three general dentists and four endodontists) on a hold-out test set.

**Results:**

The baseline VGG16 model attained 87.62% accuracy in classifying difficulty. Self-supervised pretraining did not improve performance. Regression predicted scores with ± 3.21 score error. All models outperformed human raters, with poor inter-examiner reliability.

**Conclusion:**

This pilot study demonstrated the feasibility of automated endodontic difficulty assessment via deep learning models.

## Background

Root canal treatment involves cleaning, shaping and obturation of the root canal system to prevent or treat apical periodontitis [1]. Despite relatively high success rates (82–92%) [2], endodontic treatment still carries risks of failure that can be influenced by procedural errors and mishaps [3]. Studies have demonstrated that errors including apical perforation, failing to achieve patency due to ledges or blockages, and improper obturation length can significantly reduce success rates [4–6]. This is concerning as such errors may lead to post-operative complications and tooth loss.

Anatomical complexities and aberrations in tooth crown and root canal morphology are key factors that may increase risks of procedural errors when treating difficult cases [7]. As such, appropriate pre-operative case assessment and referral of complex cases by general dentists to endodontic specialists is critically important to improve outcomes [8]. To aid standardized assessment of case difficulty, guidelines like the American Association of Endodontists’ (AAE) Endodontic case difficulty assessment categorize complexity based on multiple criteria visible on radiographs [9]. These include tooth type, arch position, rotation, extent of crown destruction, root morphology, apex diameter, and canal visibility. However, the manual application of these guidelines is time-consuming and prone to high subjectivity and interpreter variability [10, 11]. More objective automated assessment tools are needed to help general dentistry practitioners reliably gauge case difficulty from standard radiographs early on and identify cases warranting specialty referral.

Recent advances in artificial intelligence (AI), specifically deep learning, show strong promise for automating such complex diagnostic and treatment planning tasks in healthcare [12]. Deep learning utilizes multi-layered neural networks capable of automatically identifying intricate patterns and relationships in data without the need for explicit human programming. Within medicine, deep learning has already demonstrated expert-level performance analyzing medical images for various tasks. For example, deep learning models have shown accuracies rivaling healthcare specialists in diagnosing diabetic retinopathy from retinal fundus images [13] and predicting skin cancer from clinical images [14]. Within the dental field, preliminary research has applied deep learning models for tasks including tooth numbering [15, 16], caries diagnosis [17], detection of periapical lesions [18], and extraction difficulty of third molars [19, 20]. However, deep learning has traditionally relied heavily on supervised learning techniques, which require massive manually annotated datasets that are expensive, time-consuming, and prone to human subjectivity, inconsistency, and errors [21]. Transfer learning can mitigate this by initializing models with general image features learned on large datasets, before fine-tuning on more limited medical data. An alternative approach is self-supervised learning (SSL), which can pre-train neural networks on abundant unlabeled medical imaging data [22]. SSL models learn meaningful feature representations from the data itself without the need for manual labeling, which is especially valuable for specialized fields like dentistry, where annotated data is scarce. Moreover, studies have shown SSL models can surpass supervised models in disease detection from retinal images [23].

For endodontic case difficulty assessment, which involves assessing multiple anatomical factors, an SSL approach seems promising. However, no previous studies found that have investigated deep learning techniques for standardized pre-operative assessment of non-surgical endodontic treatment case difficulty. In this study, we aimed to develop and validate a diagnostic tool using deep learning on periapical radiographs to determine endodontic case difficulty based on established guidelines.

## Materials and methods

### Study design

This retrospective study utilized deep convolutional neural network models to assess endodontic case difficulty from periapical radiographs based on AAE guidelines. In our research paper, we examined five pre-trained and state-of-the-art convolutional neural network (CNN) architectures, namely ResNet50, ResNet18, Inception V2, VGG16 (with batch normalization layers), and ResNext50. Additionally, we explored four self-supervised learning (SSL) approaches, namely SimCLR, MoCo, BYOL, and DINO. All models were employed to categorize endodontic case difficulty for binary classification analysis. Additionally, the top performing model was leveraged to predict overall difficulty scores through regression. The Ethics Committee of the Hamadan University of Medical Sciences approved this study (IR.UMSHA.REC.1402.026). The study results were reported in accordance with the Checklist of Artificial intelligence in Medical imaging [24].

### Dataset and preparation

A dataset of 1,386 periapical radiographs of adult patients was compiled, including images from the radiology department of Hamadan University of Medical Sciences and a private dental clinic in Hamadan, Iran. Radiographs were captured using MINRay (Soredex, Tuusula, Finland) radiology system and Optime (Soredex, Tuusula, Finland) size #2 phosphorplate sensors. Radiographic exposure settings were standardized with a tube voltage of 60 kV, tube current of 7 mA, and exposure times ranging from 0.16 to 0.32 s adjusted based on tooth type. Inclusion criteria were permanent teeth with fully visible crowns and roots without obscuring artifacts. Exclusion criteria were deciduous teeth, impacted teeth, presence of orthodontic appliances, and poor image quality due to processing errors or patient motion or any other artifacts.

### Ground truth annotations

All images were de-identified using randomized numeric labels. The periapical images needed for the research were selected by the main researcher (S.S) and two dentists (N.G and A.M) labeled the dataset for endodontic case difficulty based on the latest AAE guidelines. Difficulty ratings of low (1 point), moderate (2 points) and high (5 points) were assigned for the following criteria: tooth type, inclination, rotation, crown anatomy, root morphology, apex diameter, and canal visibility, mirroring the scoring system used in the “simple assessment” in AAE EndoCase mobile application.

The AAE guidelines involve both subjective assessments and objective measurements. Objective measurements were performed for the following features using Digimizer software v5.4.9 (MedCalc Software, Mariakerke, Belgium): Tooth length, Inclination: Tooth angle deviation in the mesiodistal dimension and canal curvature: Angle of canal curvature measured using Schneider’s method. Apex diameter is categorized based on morphology - blunderbuss apexes were considered open (> 1.5 mm) while parallel-walled open apexes were considered “between 1 and 1.5”. For crown destruction, cuspal coverage restorations and missing cusps were considered extensive. Reference images with measurements were used to improve standardization of tooth length and diameter ratings.

To establish standardized criteria, an initial set of 100 periapical radiographs were annotated by two dentists. Their difficulty ratings were reviewed by a third senior endodontist, and any disagreements or uncertainties were discussed to reach a consensus. This process refined the assessment criteria and improved inter-rater reliability. The two dentists then independently labeled the remaining radiographs in the dataset using the finalized criteria and rubric. One researcher (S.S.) evaluated the differences in ratings between the two researchers. In cases of disagreement, two board-certified endodontists with at least 10 years of experience (H.K., and E.K) were consulted to provide the decisive rating. AAE low and moderate difficulty were combined into an easy category since low difficulty corresponds only with a few anterior and premolar cases. Cases with combined scores ≤ 10 were categorized as “easy” while scores ≥ 11 were labeled as “hard” (high difficulty) for binary classification.

### Image preprocessing

Individual teeth were cropped from the periapical radiographs by a trained dentist (S.S). Images were cropped with at least 10-pixel margin and converted to JPEG format. They were resized to size 224 × 224 for all models except Inception v2 which required 299 × 299 images.

### Model architecture

#### Baseline models

Transfer learning was used to improve model training efficiency. All models were initialized with weights pre-trained on ImageNet. By transferring knowledge from this large general image dataset, the network could focus on fine-tuning dental radiograph features rather than learning from scratch. This enabled faster convergence with less data compared to a randomly initialized model. When fine-tuning the ImageNet pre-trained models, all layers were frozen except for the batch normalization layers. This allowed adjustments to the distribution of layer inputs to better fit the dental radiograph data, while retaining the learned feature representations from ImageNet in the convolutional layers. Additional, fully connected layers were constructed on top of the base models to generate predictions for the endodontic case difficulty tasks.

#### SSL models

Self-supervised pretraining was performed using contrastive learning technique [25]. The key idea is to train models to differentiate between augmented views of the same image (positive pairs) and views from different images (negative pairs). This forces the model to learn generalized visual representations based solely on the medical image data, without using any labeled categories. Typically, two separate augmented crops are created from each unlabeled dental radiograph. An encoder processes one augmented view while a separate encoder looks at the other view. If the crops originate from the same image, the encoded representations should be pulled closer together by the model. If they are from different images, the representations should be pushed apart. By optimizing this contrastive signal across many image pairs, the model learns robust features, unconfounded by any downstream task labels. For pretraining, we leveraged a diverse dataset of 20,295 unlabeled panoramic, bitewing, and periapical radiographs from a private clinic. After unsupervised pretraining, the encoder was transferred to initialize our classification model. The pretrained features were frozen, and a classifier was trained on top using the smaller labeled dataset to categorize case difficulty.

### Training details

Models are implemented in Python using PyTorch 1.7.1 on Google Collaboratory platform. Training occurred on an NVIDIA T4 Tensor core graphics processing unit with 12GB GDDR5 VRAM, paired with an Intel Xeon processor containing two 2.2 GHz cores and 13GB of RAM. Key hyperparameters were set at a learning rate of 0.001, batch size of 4 (baseline models) and 8 (SSL models), and Adam optimizer, following a randomized search strategy. The loss function is calculated via categorical cross-entropy for the classification task and mean squared error for the regression task. Due to dataset imbalance, we applied the weighted loss function. Early stopping was used to prevent the overfitting of the model. Data were augmented using random horizontal flip, random rotation, color jitter, random affine, and TrivialAugment method [26]. TrivialAugment is an automatic augmentation method that takes an image x and a set of augmentations A as input. It then simply samples a random augmentation from A uniformly, as well as a random augmentation strength m from the range [0,30]. The sampled augmentation is applied to image x with probability m, and the augmented image is returned.

### Data partitions

Initially, 202 cropped tooth images were used for our test using a random stratified sampling method. The model was trained using 10-fold stratified cross-validation to prevent overfitting and assess generalizability. The dataset was randomly split into 10 equal folds, with each fold containing a similar distribution of easy and hard cases. For each fold, the model was trained on the other 9 folds and validated on the held-out fold. This was repeated until all folds served as the validation set once. All model hyperparameters were tuned using the 9-fold training sets only. The cross-validation results were then aggregated to evaluate model performance. Model generalizability was assessed by averaging accuracy across the 10 validation folds.

### Clinician assessment

Periapical images from the test set were provided to three general dentists and four endodontists for individual assessment separately. The examiners, comprising general dentists with an average of 2.6 years and endodontists with an average of 8 years of clinical experience, were given a brief orientation on case difficulty assessment using the simple assessment in EndoCase application. No formal calibration was performed in order to evaluate independent analysis. The evaluations were conducted under consistent controlled conditions, with images displayed on standard 1080p monitors in a darkened room to minimize external distractions and simulate ideal clinical viewing settings. Working independently, the evaluators categorized each tooth into ‘hard’ or ‘easy’ groups based on their clinical experience and judgment, guided qualitatively by the criteria outlined in the assessment tools.

### Evaluation

We evaluated models and clinician performance on a held-out test set. The accuracy, precision, recall, and F1-score of the model/clinician on the test set were presented for each class and dataset.

$$Precision = \frac{{TP}}{{TP + FP}}$$

$${\text{Recall = }}\frac{{TP}}{{TP + FN}}$$

$${\text{Accuracy = }}\frac{{TP + TN}}{{TP + TN + FP + FN}}$$

$${\text{F1 score = }}\frac{{2TP}}{{2TP + FP + FN}}$$

Where TP, TN, FP, and FN are the number of true-positive, true-negative, false-positive, and false-negative samples, respectively. Confusion matrices were generated and receiver operating characteristic (ROC) curves plotted with Area Under the Curve (AUC) metrics assessed for each model. Interobserver agreement performance was assessed using Fleiss kappa. The results were interpreted as follows: Kappa < 0.2: Slight agreement, 0.21–0.4: Fair agreement, 0.41–0.6: Moderate agreement, 0.61–0.8: Substantial agreement, 0.81: Almost perfect agreement. Statistical analyses were performed using SPSS for Windows version 15 (SPSS Inc., Chicago, IL, USA).

## Results

### Study populations

The distribution of case difficulty items is presented in Table 1. Our dataset comprises 603 molar teeth and 783 anterior/premolars.

**Table 1:** The distribution of case difficulty items

Category	Subcategory		Number of Labels
Tooth type	Anterior/Premolar:		783
	First Molar:		331
	Second Molar:		272
Inclination	Less than 10 degrees:		1195
	Between 10 to 30 degrees:		164
	More than 30 degrees:		27
Rotation	Less than 10 degrees:		1308
	10 to 30 degrees:		32
	More than 30 degrees:		46
Crown Morphology	Normal crown shape:		912
	Severe crown destruction:		474
	Taurodontism:		110
	Microdontia:		0
	Abutment bridge:		128
	Porcelain veneer:		0
	Full coverage restoration:		209
	Fusion:		0
	Dens in dente:		0
	Restoration does not reflect original anatomy:		4
Root morphology	Root curvature angle	Less than 10 degrees:	1121
		Between 10 to 30 degrees:	191
		More than 30 degrees:	74
	Crown and root axis substantially different:		26
	Radix ento/paramolaris:		62
	Long tooth (longer than 30 mm):		79
	Canal splits in middle or apical third:		44
	Maxillary premolar with 3 roots:		0
	Mandibular anterior or premolar with 2 roots:		91
	S-shaped canal curvature:		48
	C-shaped morphology:		98
Apex Diameter	Closed (less than 1 mm):		1294
	Between 1 to 1.5 mm:		46
	Open (more than 1.5 mm):		46
Canal and pulp chamber	Visible/not reduced:		1301
	Visible/reduces:		286
	Not visible:		69
	Pulp Stones:		136

### Classification task

**Fig. 1:**
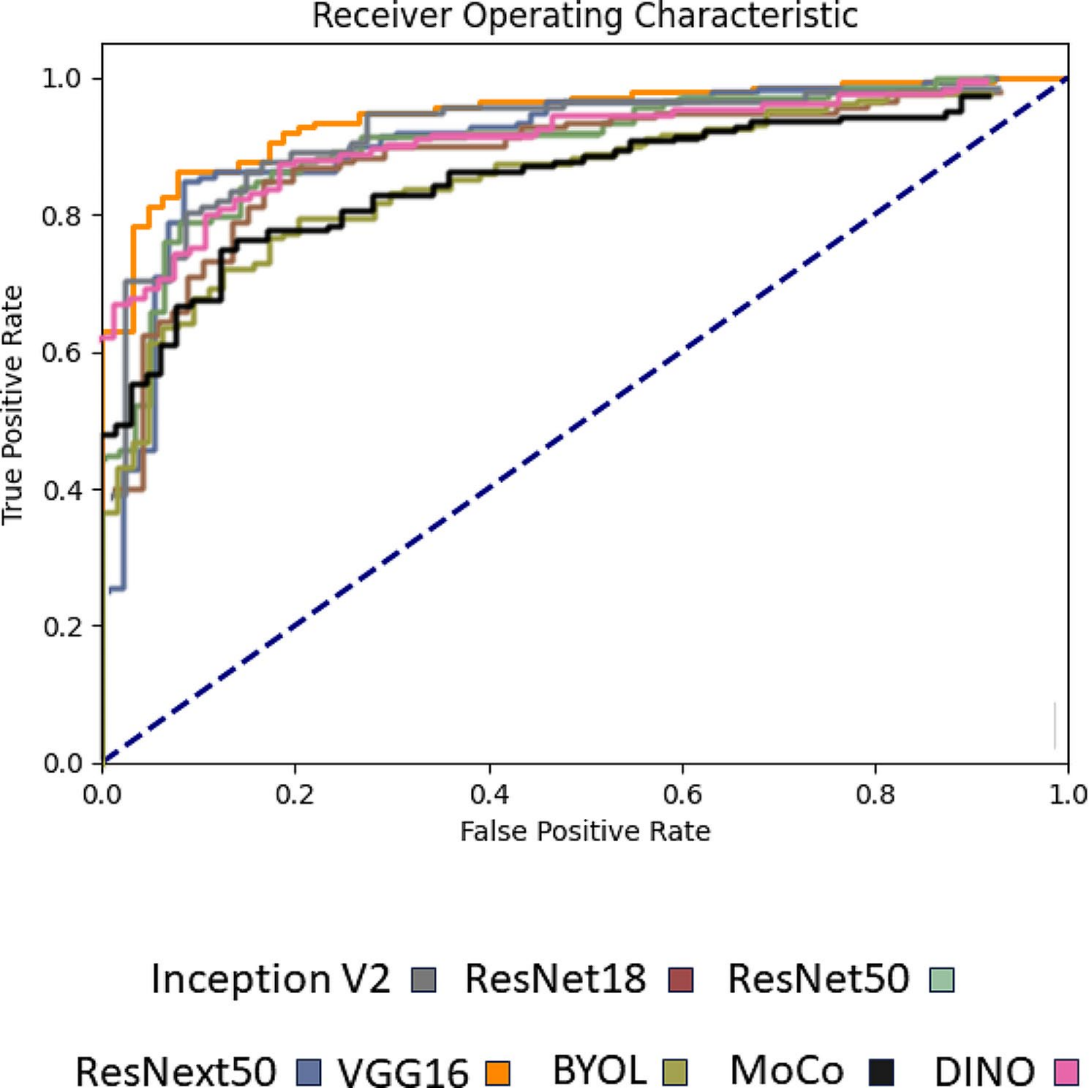
Receiver Operating Characteristic (ROC) curves illustrating the prediction performance of models on the test set, with each model represented by a distinct color

The results from 10-fold cross validation, accuracy, precision, recall, and F-1 score are presented in Table 2. Additionally, ROC curves of models are shown in Fig. 1. Inception v2 and DINO models had the best cross-validation accuracy, at 91.05% and 91.04%, respectively. VGG16 and Inception v2 models had the best AUC score, at 94.36% and 92.42%, respectively. VGG16 model had the best overall precision, recall, and accuracy across all models.

**Table 2:** The results from cross validation accuracy and precision, recall, and F1-score of all models in the test set

Model Name	Cross validation	Test					
	Average accuracy	AUC Score	Accuracy	Class	Precision	Recall	F1 score
VGG16	89.11	94.36	87.62	Easy	75.32	90.62	82.26
				Hard	95.2	86.23	90.49
				Weighted Average	88.9	87.6	87.8
ResNet18	89.19	89.07	84.65	Easy	72.00	84.37	77.69
				Hard	92.12	84.78	88.30
				Weighted Average	85.7	84.6	84.9
ResNet50	90.80	90.62	85.15	Easy	73.61	82.81	77.94
				Hard	91.53	86.23	88.80
				Weighted Average	85.85	85.14	85.35
ResNext50	90.71	91.34	84.16	Easy	76.66	71.87	74.19
				Hard	87.32	89.85	88.57
				Weighted Average	83.94	84.15	84.01
Inception v2	91.05	92.42	86.14	Easy	81.03	73.43	77.04
				Hard	88.19	92.02	90.07
				Weighted Average	85.92	86.13	85.94
BYOL	83.94	86.95	79.70	Easy	65.3	76.56	70.50
				Hard	88.18	81.15	84.52
				Weighted Average	80.93	79.69	80.07
SimCLR	87.16	91.16	82.67	Easy	69.33	81.25	74.82
				Hard	90.55	83.33	86.79
				Weighted Average	83.82	82.67	82.99
MoCo v2	82.76	85.71	79.21	Easy	63.09	82.81	71.62
				Hard	90.67	77.53	83.59
				Weighted Average	81.93	79.20	79.79
DINO	91.04	91.07	85.15	Easy	74.28	81.25	77.61
				Hard	90.90	86.95	88.88
				Weighted Average	85.63	85.14	85.30

**Fig. 2:**
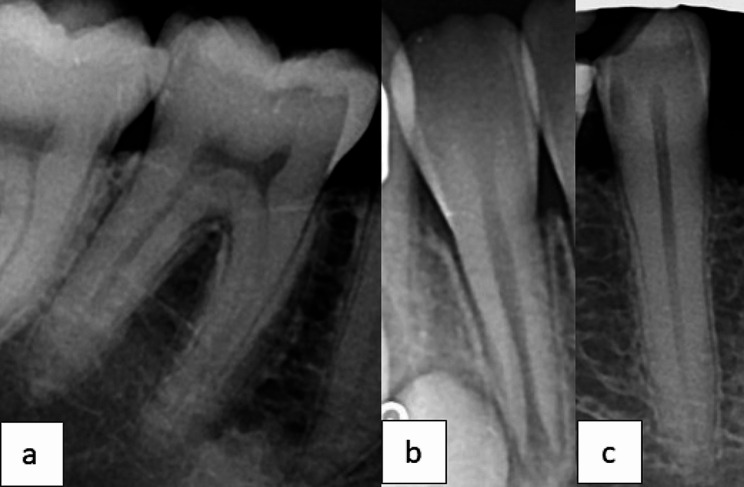
Error samples in VGG16 predictions. **a**, reduced canal **b**, open apex **c**, long tooth

In Fig. 2 error samples of VGG16 model are illustrated. Error analysis showed that false predictions in “Easy” category predominantly occurred in teeth with reduced canals. False predictions in the “Hard” category were mainly associated with anterior tooth with open apex and long tooth.

### Regression task

Given its superior classification performance, the VGG16 model was selected for the regression task. The average mean squared error was recorded at 10.32. The model can predict the difficulty score with an error margin of ± 3.21.

### Clinician assessment

The evaluation metrics of human performance is provided in Table 3. All human scores were lower than deep learning models. The Fleiss Kappa interobserver agreement between all groups, general dentists and endodontists were 0.22, 0.54, and 0.39, respectively. General dentists have moderate agreement but the level of agreement between the endodontists and all observers is fair.

**Table 3:** Human performance on the test set

Human Performance	Years of Experience	Class	Test		
			Precision	Recall	F1-score
General dentist 1	4	Easy	59.09	81.25	68.42
		Hard	89.47	73.91	80.95
		Weighted Average	79.84	76.23	76.98
General dentist 2	2	Easy	53.51	79.68	62.57
		Hard	87.37	65.21	74.68
		Weighted Average	76.64	69.79	70.84
General dentist 3	2	Easy	48.23	64.06	55.03
		Hard	80.34	68.11	73.72
		Weighted Average	70.16	66.82	67.79
Endodontist 1	6	Easy	53.68	79.68	64.15
		Hard	89.52	68.11	76.73
		Weighted Average	78.16	71.77	72.74
Endodontist 2	9	Easy	59.37	66.66	62.80
		Hard	86.23	82.06	84.09
		Weighted Average	77.71	77.18	77.34
Endodontist 3	8	Easy	44.73	79.68	57.30
		Hard	85.22	54.34	66.37
		Weighted Average	72.39	67.01	61.83
Endodontist 4	9	Easy	75.00	48.43	57.40
		Hard	79.11	90.57	84.59
		Weighted Average	77.8	77.21	75.97
Average			76.1	72.28	71.92

## Discussion

Assessment of case complexity remains a key challenge in endodontic diagnosis and treatment planning, requiring comprehensive evaluation through clinical examination, radiographic analysis, and understanding of the operator’s skills [1]. Attempting procedures beyond one’s capabilities risks intraoperative errors and subsequent harm to patient health, while also exposing clinicians to potential legal repercussions [8]. Thus, guidelines have been developed to determine case difficulty for procedures like molar extraction and root canal therapy, aiding clinical decision-making. Our present study demonstrates for the first time that deep learning models can predict endodontic treatment difficulty from periapical radiographs with high accuracy.

Deep learning has garnered considerable interest in medicine owing to its high learning capacity and demonstrated ability to automate intricate diagnostic and treatment planning tasks. In endodontics, deep learning has shown promise in detecting vertical root fracture [3] and special tooth anatomies like C-shaped canals [27], and taurodontism [28]. They may also assist in gauging treatment complexity to inform planning and referral needs. For instance, CNNs have been applied to categorize third molar surgery difficulty [29]. We examined several state-of-the-art convolutional neural network architectures for this classification task. Among the supervised models examined, Inception v2 achieved the highest cross-validation accuracy at 91.05%, while VGG16 demonstrated the best overall test performance - attaining 87.62% accuracy, 94.36% AUC, with the top precision, recall and F1-scores. The self-supervised DINO model narrowly exceeded VGG16 in cross-validation accuracy, while most other SSL techniques failed to match these top supervised models. While self-supervised pretraining demonstrates promise for medical imaging tasks, it did not improve overall accuracy in this study compared to supervised training alone. Error analysis revealed the majority of model errors occurred in cases with reduced canal visibility, long tooth lengths, and open apices. These features were severely underrepresented in the dataset, indicating that SSL may still be advantageous given sufficient sample diversity. Nevertheless, all deep learning models showed higher precision and recall than human raters. The low inter-rater agreement highlights issues with consistency using the AAE guideline. Automated AI assessment could address these reliability limitations while improving accuracy.

For root canal therapy, the AAE case difficulty assessment is widely utilized, and had proved useful in predicting endodontic mishaps [30], obturation length [1] and 4-year clinical success rate [31]. It categorizes cases as “low difficulty,” “moderate difficulty” or “high difficulty” based on both patient-related and tooth-related factors. However, we only assessed simple tooth related factors that can be assessed on periapical radiographs using “simple assessment” in AAE’s EndoCase application. We combined low and moderate difficulty into “easy” category and high difficulty into “hard” category for our classification analysis, since low difficulty only possess a very few cases of anterior and premolar teeth. The present study is the first time to incorporate deep learning in endodontic case difficulty assessment. By developing an objective, computational method, we aimed to address longstanding issues with subjectivity in conventional human-based assessments. Our model could help standardize case selection for dental students and junior clinicians by supplementing evaluation with a data-driven approach.

In addition to classification, we applied the model for regression to predict individual difficulty scores utilizing the scoring system of AAE’s EndoCase application. This scoring system developed by endodontic specialists categorizes complexity on a scale starting at 7. VGG16 predicted scores to within ± 3.21 units. Predicting the overall difficulty level rather than a binary label may offer useful clinical insights. Higher scores could flag challenging cases requiring more appointment time or specialist referral to reduce clinician fatigue and improve outcomes. With further validation, AI-predicted difficulty scoring could potentially assist in balanced pre-operative case scheduling. Further studies are needed to assess the clinical significance of difficulty scores. Moreover, there is a need to develop AI-optimized rating guidelines, better suited to algorithmic analysis. Retooling for AI compatibility could improve assessment standardization.

This study had some limitations. By performing binary classification rather than predicting specific difficulty factors, some diagnostic detail was lost. Additionally, periapical radiographs provide limited two-dimensional information compared to 3D modalities like cone-beam CT. Assessing complex anatomical factors in 3D teeth using 2D images presents challenges. For example, accurate characterization of canal curvatures, divisions and root morphologies like radix ento/paramolaris is difficult without clear 3D visualization. Furthermore, clinical examination is imperative for comprehensive assessment of patient-specific factors as well as tooth inclination and rotation. Moving forward, integrating three-dimensional imaging and patient record details could enhance modeling capabilities. Future work should also aim to elucidate individual tooth characteristics driving treatment complexity.

## Conclusion

This pilot investigation highlights the promise of deep learning to automate endodontic difficulty assessment as a clinical decision support tool. With further refinements to models and data sources, such an approach could potentially help standardized preoperative evaluation.

## Data Availability

The datasets used and/or analyzed during the current study available from the corresponding author on reasonable request.
